# Translation of the humeral head scale is associated with success of rotator cuff repair for large-massive tears

**DOI:** 10.1186/s12891-017-1874-9

**Published:** 2017-12-04

**Authors:** Noboru Taniguchi, Darryl D. D’Lima, Naoki Suenaga, Yasuyuki Ishida, Deokcheol Lee, Isoya Goya, Etsuo Chosa

**Affiliations:** 1 Department of Orthopaedic Surgery, University of Miyazaki, 5200 Kihara, Kiyotake, Miyazaki, 889-1692 Japan; 2 Department of Medical Science, Tokyo Medical University, 6-1-1 Shinjuku, Shinjuku-ku, Tokyo, 160-8402 Japan; 3 Shiley Center for Orthopaedic Research and Education, Scripps Clinic, 11025 North Torrey Pines Road, Suite 200, La Jolla, CA 92037 USA; 4 Department of Molecular Medicine, The Scripps Research Institute, 10550 North Torrey Pines Road, La Jolla, CA 92037 USA; 5 The Upper Extremity Center of Joint Replacement and Endoscopic Surgery, Hokushin Orthopaedic Hospital, 1-18 Kikusuimotomachi 3-jou 3-chome, Shiroishi-ku, Sapporo, 003-0823 Japan; 6 Hakuaikai Kaisei Hospital, 16-27 Nishi-23 Minami-2, Obihiro, Hokkaido 080-2473 Japan

**Keywords:** Large and massive rotator cuff tears, Anterolateral migration of the humeral head, Translation of the humeral head scale, Acromiohumeral interval, Cuff repair integrity

## Abstract

**Background:**

Although a loss of rotator cuff integrity leads to the superior migration of the humeral head, the parameters that characterize the anterolateral migration of the humeral head have not been established. The purpose of this study was to investigate the correlation between the translation of the humeral head scale (T-scale) and clinical outcomes of rotator cuff repair, as well as the correlation between the T-scale and radiologic parameters.

**Methods:**

One hundred thirty-five consecutive patients with full-thickness rotator cuff tears underwent primary rotator cuff repair. The T-scale, which indicates the distance from the center of the humeral head to the lateral coracoacromial arch, was measured on axial computed tomography scans, and the acromiohumeral interval (AHI) was measured radiographically. The correlation of the two parameters with the clinical scores of the Japanese Orthopaedic Association and University of California–Los Angeles scores and active forward elevation (FE) were evaluated at the preoperative and postoperative stages, respectively.

**Results:**

The postoperative T-scale and AHI correlated well with the postoperative FE and clinical scores in the patients with large-massive tears but not in those patients with small-medium tears and preoperative large-massive tears. A significant correlation was observed between the postoperative T-scale and AHI. The T-scale was subject to cuff repair integrity.

**Conclusions:**

We demonstrated that the postoperative T-scale was well correlated with the clinical results and postoperative AHI after rotator cuff repair for large-massive tears, indicating that poor outcomes are associated with combined superior and anterolateral migration of the humeral head following retears.

## Background

Patients with large-massive rotator cuff tears present a surgical challenge to the relief of pain and restoration of shoulder function. A loss of rotator cuff integrity creates an unstable fulcrum of motion, leading to the superior migration of the humeral head on the glenoid and altered glenohumeral joint biomechanics [1]. Various techniques are recommended to address such tears, including debridement with acromioplasty, partial repair, primary arthroscopic repair, mini-open repair, tissue augmentation, tendon transfer, hemiarthroplasty, and reverse shoulder arthroplasty [2–11]. However, many large-massive tears are associated with high rates of recurrent tendon defects on follow-up examinations [4, 12], which are associated with postoperative strength deficits and poorer outcome scores [13, 14].

Irreparable rotator cuff deficiencies and coracoacromial arch losses are complicated by the compromise of the anterior portion of the deltoid muscle as a result of surgical procedures, particularly in patients with a prior open repair [15]. The intact posterior and lateral portions of the deltoid exert a net posterior vector on the mid-shaft of the humerus during contraction, which intensifies the anterior escape of the humeral head. Anterosuperior escape may also be exacerbated by asymmetric posterior capsular contracture. The combined loss of the rotator cuff, coracoacromial arch, and anterior deltoid integrity results in dynamic anterosuperior instability that occurs during attempted elevation [16].

The acromiohumeral interval (AHI) has been proposed as a reliable measure of superior migration of the humeral head [17]. Using this scale, Hamada et al. radiographically classified massive rotator cuff tears into five grades [18] and suggested that cuff repair should be performed before AHI narrowing occurs [19]. The Seebauer classification system provides a biomechanical description of rotator cuff tear arthropathy and distinguishes each type of tear based on the degree of superior migration from the center of rotation and the amount of instability [20]. However, the distinction between type 2A and 2B lesions, which is based on the integrity of the anterior restraints and structures, is difficult to assess on plain radiographs; moreover, the parameter that elucidates the anterolateral migration of the humeral head has yet to be established.

We recently developed a novel parameter to measure the anterolateral migration of the humerus: the translation of the humeral head scale by Taniguchi (T-scale) [21]. The purpose of this study was to investigate the correlation between the T-scale and clinical outcomes following rotator cuff repair and the correlation between the T-scale and radiologic parameters. We hypothesized that this scale might explain significant postoperative differences and could potentially serve as a prognostic factor to measure the success of rotator cuff repair.

## Methods

### Patient selection

Ethical committee approval for the present study was obtained from the University of Miyazaki Ethics Board. This case series study (level of evidence IV) consisted of 135 consecutive patients with full-thickness rotator cuff tears who failed to undergo conservative treatment and underwent primary cuff repair between January 2010 and August 2015. Twelve patients were excluded because computed tomography (CT) scans were not available, and 15 patients were lost to follow up. The remaining 108 patients (37 women and 71 men) had a mean age of 65.1 years (range, 40–86 years) at the time of surgery. The rotator cuff tears were diagnosed primarily by physical examination, and T2-weighted spin-echo magnetic resonance imaging (MRI) was performed for all patients preoperatively and at the last follow-up visit. The standardized follow-up time points were at 1 year or more after surgery. Patients were excluded if they had a history of dislocation or fracture of the shoulder, degenerative or inflammatory arthritis, infection, neuropathic changes, prior surgical procedures on the shoulder.

### Operative techniques

During the surgery, acromioplasty was performed by resection of the coracoacromial ligament according to the method described by Ellman [22]. The tear size was measured intraoperatively and classified using the system described by DeOrio and Cofield as small (<1 cm), medium (1 cm to 3 cm), large (3 cm to 5 cm), and massive (>5 cm, or involving two tendons) [23]. Patients with small-medium tears underwent arthroscopic single-row repair, the suture bridge method, or the surface-holding method [24–26], and patients with large-massive tears underwent arthroscopic suture bridge and the surface-holding method or open surface-holding repair [27]. The surface-holding method is a modified transosseous-equivalent procedure using medial anchors and lateral transosseous sutures [26, 28], and in certain cases, the bone marrow stimulation technique was applied after anchor insertion [29]. During postoperative rehabilitation, an abduction pillow was used for six weeks in cases of small-medium tears and for eight weeks in cases of large-massive tears [26, 29].

### Clinical assessment

All patients underwent a physical examination prior to surgery. Postoperative evaluations were performed at 1 year or more after surgery, and the Japanese Orthopaedic Association (JOA) shoulder score (100-point scoring system) [30] and University of California–Los Angeles (UCLA) rating scale (35-point scoring system) [17] were recorded at each time point.

### Radiographic assessment

Preoperatively and at the last follow-up visit, a true anteroposterior radiograph in neutral shoulder rotation with the patient standing was obtained, and the AHI was assessed in accord with the method of Iannotti et al. [31]. In cases of superior escape in which the line tangent to the top of the humeral head was higher than the line parallel to the undersurface of the acromion, the AHI was represented as a negative value. The patients were assessed using axial CT scans at the preoperative stage and the latest follow-up visit.

**Fig. 1:**
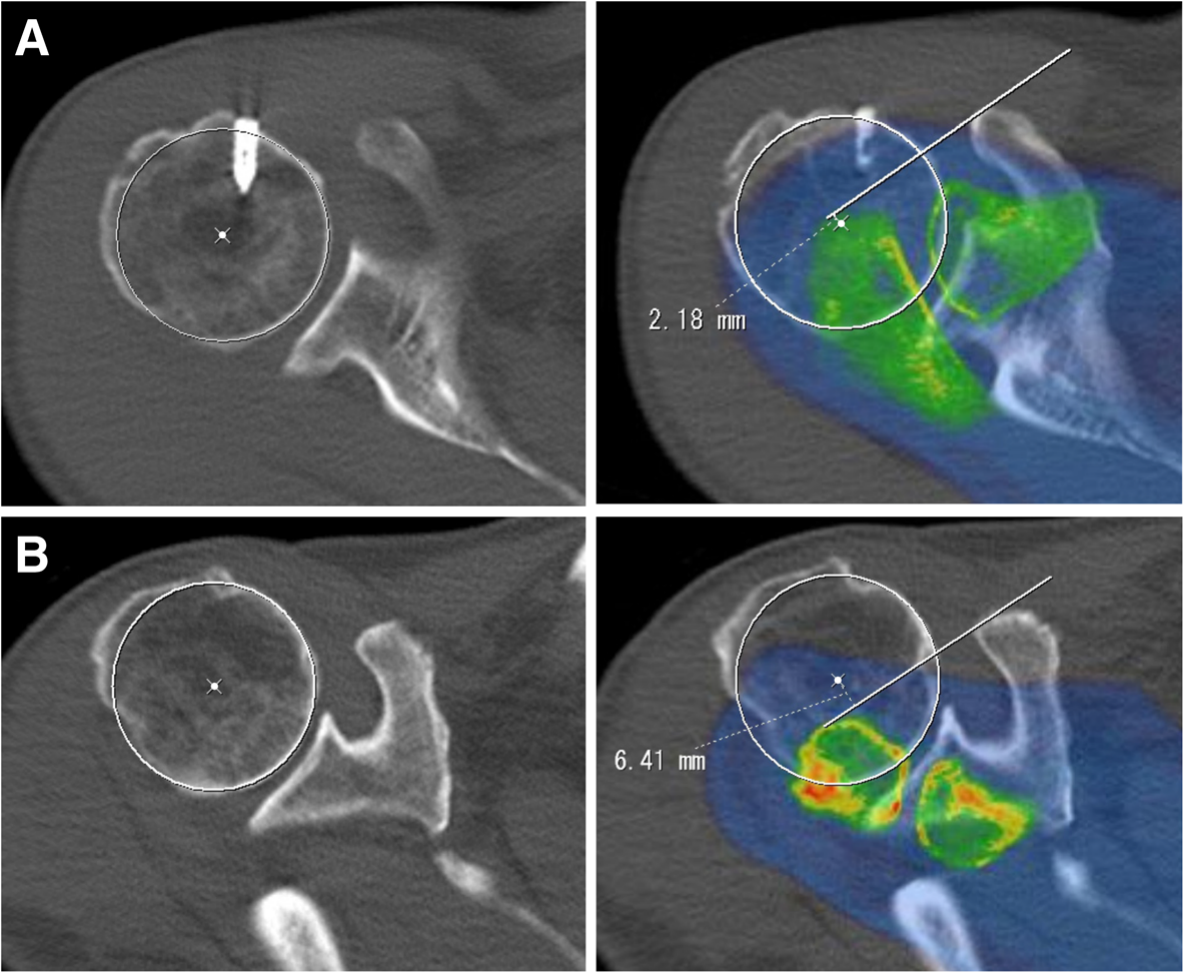
T-scale measurements. Using merged slices of the anterolateral edge of the acromion and the lateral edge of the coracoid process, a line was drawn between the two portions, and then the perpendicular distance from the center of the humeral head to the line was measured. When the center point was located outside the line, the T-scale was represented as a negative value. The measured T-scale was (**a**) 2.2 mm and (**b**) -6.4 mm

With patients in the supine position, the shoulder was maintained with the long axis of the scapular body and the long axis of the humeral shaft nearly parallel to each other, and CT scans were taken at right angles perpendicular to the long axis of the scapular body. The T-scale was measured following the method described previously [21]. The T-scale was measured using merged slices of the anterolateral edge of the acromion and the lateral edge of the coracoid process on the axial view, and a line was drawn between the two portions. Then, using the slice of the maximum diameter of the humeral head, a circle was fit to the curvature of the articular surface, and the center point was defined. Finally, the perpendicular distance from the center point to the line was measured. When the center point was located inside the line, the distance was represented as a positive value (Fig. 1a), whereas when the center point was located outside the line, the distance was represented as a negative value (Fig. 1b). The rotator cuff serves as the primary restraint to anterosuperior migration of the humeral head, while the coracoacromial arch provides a secondary restraint [16]. A line drawn between the anterolateral edge of the acromion and the lateral edge of the coracoid process represents the lateral coracoacromial arch, and the T-scale indicates the location of the humeral head relative to the coracoacromial arch.

The patients also underwent T2-weighted spin-echo MRI to evaluate the cuff repair integrity, which was classified into 5 types based on the oblique coronal and oblique sagittal views following Sugaya’s classification [30].

### Statistical analysis

Numerical data were statistically analyzed using GraphPad Prism 5.0 software (San Diego, CA, USA). The results are presented as the means and standard deviations. All data were tested for a normal distribution using the Kolmogorov–Smirnov test. Statistical comparisons between the two groups were performed using the Mann-Whitney U test and Wilcoxon signed rank test for unpaired groups and paired groups, respectively, and the chi-square test was used for categorical variables. The Spearman correlation coefficient was used to test relationships between variables. Values of *P <* 0.05 were considered significant.

## Results

The average follow-up period was 15.0 months (range, 12–40 months). The preoperative epidemiologic data for the entire population (*N* = 108) and each group (small-medium tears, *N* = 56; large-massive tears, *N* = 52) are shown in Table 1. The results indicated that age, gender, affected side, and follow-up period were similar between the small-medium tear group and large-massive tear group. At the postoperative stage, a significant increase was observed in the JOA score (*P* < 0.0001), UCLA score (*P* < 0.0001), and active forward elevation (FE) range (*P* < 0.0001) in both the small-medium tear and large-massive tear groups and for the entire group of shoulders studied (Table 2). Although the postoperative mean JOA score, UCLA score, and FE range in the large-massive tear group were significantly lower than those in the small-medium tear group (*P* < 0.0001), such differences were not found between the two groups preoperatively.

**Table 1:** Patient demographics. Data are expressed as the number of patients (%) or as the mean ± standard deviation of the mean

Variable	Total (*N* = 108)	Small/Medium (*N* = 56)	Large/Massive (*N* = 52)	*P* value
Age *(yr)*	65.1 ± 9.1	64.4 ± 8.1	65.8 ± 9.9	0.3339
Gender				0.7409
Male	71	36 (50.7%)	35 (49.3%)	
Female	37	20 (54.1%)	17 (45.9%)	
Side				0.2760
Right	72	40 (55.6%)	32 (44.4%)	
Left	36	16 (44.4%)	20 (55.6%)	
Follow-up *(mo)*	15.0 ± 6.0	15.4 ± 5.8	14.6 ± 6.2	0.4855

Patient demographics

**Table 2:** Preoperative and postoperative data for all shoulders, the small-medium tear group, and the large-massive tear group. JOA, Japanese Orthopaedic Association; UCLA, University of California–Los Angeles rating scale; FE, forward elevation. Data are expressed as the number of patients (%) or as the mean ± standard deviation of the mean

Variable	Preop.	Postop.	*P* value
JOA score *(pts)*			
Total	64.8 ± 11.6	90.5 ± 9.3	< 0.0001
Small/Medium	65.6 ± 10.0	95.5 ± 4.5	< 0.0001
Large/Massive	64.0 ± 12.9	85.0 ± 9.9	< 0.0001
*P* value	0.4531	< 0.0001	
UCLA score *(pts)*			
Total	16.0 ± 4.1	27.0 ± 5.5	< 0.0001
Small/Medium	15.9 ± 3.6	32.5 ± 2.6	< 0.0001
Large/Massive	16.0 ± 4.6	25.4 ± 5.0	< 0.0001
*P* value	0.9127	< 0.0001	
FE *(degrees)*			
Total	112 ± 42	148 ± 28	< 0.0001
Small/Medium	119 ± 38	158 ± 9	< 0.0001
Large/Massive	104 ± 45	138 ± 36	< 0.0001
*P* value	0.0509	< 0.0001	

Preoperative and postoperative data for all shoulders, the small-medium tear group, and

the large-massive tear group

### Correlation of the T-scale with the FE and clinical scores

We examined the relationship between the T-scale and FE at the preoperative and postoperative stages for both the small-medium and large-massive tears. For the small-medium tear patients, significant correlations were not observed between the T-scale and FE in either the preoperative (*r* = −0.2097, *P* = 0.1209) or postoperative measurements (*r* = −0.0589, *P* = 0.6662) (Table 3). For patients with large-massive tears, the correlation was not significant between the preoperative FE and preoperative T-scale (*r* = −0.0316, *P* = 0.8242); however, a strong positive correlation was observed between the postoperative FE and the postoperative T-scale (*r* = 0.5412, *P* < 0.0001).

**Table 3:** Correlation between the T-scale and FE/clinical scores at the preoperative and postoperative stages in small-medium tears and large-massive tears. In small-medium tears, significant correlations were not observed between the T-scale and FE at either the preoperative (*P* = 0.1209) or postoperative stages (*P* = 0.6662). In large-massive tears. a significant correlation was observed between the T-scale and FE postoperatively (*P* < 0.0001), although the correlation was not significant preoperatively (*P* = 0.8242). The JOA score (*P* < 0.0001) and UCLA score (*P* = 0.0080) were associated with the postoperative T-scale

		preoperative stage		postoperative stage	
		Correlation Coefficient	*P* Value	Correlation Coefficient	*P* Value
Small-Medium tears	FE	−0.2097	0.1209	−0.0589	0.6662
	JOA	−0.2378	0.0776	−0.2018	0.2525
	UCLA	−0.2138	0.1170	−0.2466	0.3755
Large-Massive tears	FE	−0.0316	0.8242	0.5412	< 0.0001
	JOA	0.1357	0.3374	0.5136	< 0.0001
	UCLA	−0.0222	0.8759	0.3638	0.0080

Correlation between the T-scale and FE/clinical scores at the preoperative and postoperative stages in small-medium

tears and large-massive tears

Next, we investigated whether this correlation was reproducible in the clinical scores. We observed significant correlations between the postoperative T-scale and the postoperative JOA score (*r* = 0.5136, *P* < 0.0001) and between the postoperative T-scale and the postoperative UCLA score (*r* = 0.3638, *P* = 0.0080). No significant correlations were found between the preoperative T-scale and the preoperative JOA score (*r* = 0.1357, *P* = 0.3374) or between the preoperative T-scale and the preoperative UCLA score (*r* = −0.0222, *P* = 0.8759). In summary, these findings indicate a strong association of the postoperative T-scale with the postoperative FE and with the postoperative clinical scores in large-massive tear patients. The representative data of patients treated with cuff repair for massive rotator cuff tear are shown in Fig. 1. At the postoperative stage, the patient with a T-scale of +2.2 mm (Fig. 1a) presented a JOA score, a UCLA score and an FE of 89.5 points, 30 points and 150 degrees, respectively, whereas in the patient with a T-scale of −6.4 mm (Fig. 1b), these values were 67 points, 17 points and 45 degrees, respectively.

### Correlation of AHI with the FE and clinical scores

Next, we measured the conventional AHI using the radiographs of the patients with small-medium tears and large-massive tears. We did not observe a significant correlation for either small-medium tears or large-massive tears between the preoperative AHI and the preoperative FE (*r* = −0.1133, *P* = 0.4058; *r* = 0.070, *P* = 0.5876), between the preoperative AHI and the preoperative JOA score (*r* = −0.1170, *P* = 0.3905; *r* = 0.2100, *P* = 0.1352) or between the preoperative AHI and the preoperative UCLA score (*r* = −0.1613, *P* = 0.2393; *r* = 0.1533, *P* = 0.2778) (Table 4). However, a significant linear relationship was observed between the postoperative AHI and the postoperative FE in patients with large-massive tears (*r* = 0.5170, *P* < 0.0001). A significant positive correlation was also found when the postoperative AHI was compared with the postoperative JOA score (*r* = 0.5567, *P* < 0.0001) and the postoperative UCLA score (*r* = 0.2841, *P* = 0.0413), although that correlation was not observed in patients with small-medium tears postoperatively.

**Table 4:** Correlation between the AHI and FE/clinical scores at the preoperative and postoperative stages in small/medium tears and large-massive tears. At the preoperative stage, no significant correlation was observed between the AHI and FE in both small-medium tears and large-massive tears, whereas at the postoperative stage, a significant linear correlation was found between the AHI and FE (*P* < 0.0001), between the AHI and JOA score (*P* < 0.0001), and between the AHI and UCLA score (*P* = 0.0413) in large-massive tears

		preoperative stage		postoperative stage	
		Correlation Coefficient	*P* Value	Correlation Coefficient	*P* Value
Small-Medium tears	FE	−0.1133	0.4058	0.0857	0.5302
	JOA	−0.1170	0.3905	0.1252	0.3579
	UCLA	−0.1613	0.2393	0.2430	0.3829
Large-Massive tears	FE	0.0770	0.5876	0.5170	< 0.0001
	JOA	0.2100	0.1352	0.5567	< 0.0001
	UCLA	0.1533	0.2778	0.2841	0.0413

Correlation between the AHI and FE/clinical scores at the preoperative and postoperative stages in small/medium tears and large-massive tears

### Correlation of the T-scale with the AHI

Because both the T-scale and AHI were well correlated with the FE range and the clinical scores postoperatively, we sought to examine the relationship between the T-scale and the AHI. At the preoperative stage, significant correlations were not observed between the T-scale and AHI (*r* = −0.2127, *P* = 0.1300); however, at the postoperative stage, a significant linear correlation was found between the two parameters (*r* = 0.4330, *P* = 0.0013) (Table 5). These findings demonstrated that the postoperative T-scale and the postoperative AHI converged after repair of large and massive tears.

**Table 5:** Correlation between the T-scale and AHI. At the preoperative stage, no significant correlation was observed between the T-scale and AHI (*P* = 0.1300), whereas at the postoperative stage, a significant linear correlation was observed between the two parameters (*P* = 0.0013)

preoperative		postoperative	
Correlation Coefficient	*P* Value	Correlation Coefficient	*P* Value
−0.2127	0.1300	0.4330	0.0013

Correlation between the T-scale and AHI

### Postoperative T-scale and cuff repair integrity

Because cuff integrity can affect clinical and functional results even several years after rotator cuff repair [32], we compared the postoperative T-scale and the postoperative AHI with cuff repair integrity and found that the postoperative T-scale (*r* = −0.3146, *P* = 0.0231) and the postoperative AHI (*r* = −0.3137, *P* = 0.0235) were both significantly correlated with cuff repair integrity (Table 6). These findings suggest that the anterolateral migration of the humeral head after rotator cuff repair, which was indicated by the postoperative T-scale, was subject to cuff repair integrity.

**Table 6:** Correlation between the postoperative T-scale/AHI and cuff integrity. The T-scale (*P* = 0.0231) and AHI (*P* = 0.0235) were both significantly correlated with cuff repair integrity at the postoperative stage

	postoperative T-scale		postoperative AHI	
	Correlation Coefficient	*P* Value	Correlation Coefficient	*P* Value
Cuff integrtiy	−0.3146	0.0231	−0.3137	0.0235

Correlation between the postoperative T-scale/AHI and cuff integrity

## Discussion

The AHI is commonly used to grade the severity of cuff tears [18] but does not directly measure the integrity of the anterior restraints and structures. The humeral head migrates anterolaterally after loss of the coracoacromial arch, weakness or insufficiency of the anterior deltoid, and asymmetric contracture of the posterior capsule. This anterolateral migration of the humeral head is difficult to assess on plain radiographs. We have proposed a novel, clinically relevant parameter (T-scale) for measuring the anterolateral migration of the humeral head in patients with rotator cuff tears. The postoperative T-scale correlated well with the clinical outcomes and integrity of rotator cuff repair for patients with large-massive tears.

A decreasing or negative T-scale represents anterolateral migration of the center of the humeral head beyond the line of the lateral coracoacromial arch, indicating loss of the secondary restraint provided by the coracoacromial arch. Although AHI is commonly used to classify the severity of rotator cuff deficits, studies have shown that radiographs to determine the acromial shape does not have high intra- or inter-observer reliability [33–35]. AHI may be confounded by acromial morphology or acromial osteophytes, making its measurement less objective and reproducible, particularly after treatment with acromioplasty. The shape of the acromion is also affected by the angle of the radiograph, so subtle changes in the radiological beam can change the perceived shape of the acromion. AHI also does not directly measure the component of anterior translation in the case of anterosuperior escape of the humerus. Furthermore, measurements of AHI on MRI or CT scans obtained in the supine position are typically smaller than conventional radiographic measurements [36, 37]. We therefore sought an alternate measure of anterolateral instability that was reproducible and had predictive potential.

We examined the relationship between the T-scale and FE at the preoperative and postoperative stages for both small-medium tears and large-massive tears. At the preoperative stage, the data were evenly distributed for both the small-medium and large-massive tears without a significant correlation between the two parameters; however, at the postoperative stage, a strong positive correlation existed between the T-scale and FE in large-massive tears. Moreover, a significant correlation was observed between the T-scale and the clinical scores. These findings indicate that the humeral head had tended to shift anterolaterally in the patients with poor surgical outcomes. This correlation indicates that the surgical technique likely affected the stability of glenohumeral joint, and re-establishing the appropriate force couples is important for obtaining a stable fulcrum for humeral head rotation.

Regarding the AHI in the patients with large-massive tears, a significant positive correlation was observed with the FE and clinical scores postoperatively. This result is consistent with a previous report showing that the postoperative AHI correlated significantly with the postoperative Constant score, size of the re-rupture, and postoperative fatty degeneration of the infraspinatus [13]. Our results demonstrated that both the T-scale and the AHI correlated well with the FE and clinical scores postoperatively and that the T-scale and AHI were significantly and positively correlated at the postoperative stage. A loss of rotator cuff integrity leads to the superior migration of the humeral head on the glenoid [1]. In our hands, the T-scale and AHI were well correlated with cuff repair integrity. These findings suggest that rotator cuff re-tearing may be responsible for superior migration and anterolateral migration of the humeral head. It is also possible that the T-scale measured at an early postoperative time point might be an early marker of failure of rotator cuff repair for large-massive tears. Acromioplasty can affect the postoperative AHI, but may have a smaller effect on the T-scale because the anterolateral edge of the acromion is relatively preserved.

This study had some limitations. Because the T-scale is subject to the morphology of the acromion and coracoid process, anatomical variations among individuals may influence this factor. The coracoacromial ligament was transected in all cases, which may cause increased anterosuperior escape in patients with large-massive cuff tears, and the different surgical techniques for both small-medium and large-massive tears may affect the clinical results. There is possibility of inter-observer and intra-observer variability in placing a line between the anterolateral edge of the acromion and the lateral edge of the coracoid process. Our intercorrelation coefficients were acceptable at 0.89 and 0.93, respectively [21]. Postoperatively, both the AHI and T-scale were measured at the same time as the clinical assessment. Therefore, one cannot determine a cause-and-effect relationship between T-scale and failure of cuff repair. Only FE was recorded, not abduction, internal rotation or external rotation. Additionally, the follow-up period was a minimum of 1 year; however, the postoperative shoulder active range of motion plateaus at 6 months, and the clinical scores do not change significantly after 1 year [38].

## Conclusions

We demonstrated that the T-scale was well correlated with the clinical results of rotator cuff repair and the AHI for large-massive tears. Our results indicate that poor outcomes after repair, especially with retears of the cuff, are associated with combined superior and anterolateral migration of the humeral head. It is also possible that early postoperative T-scale measurements could be an early marker of clinical outcomes, which might be useful to more closely follow up at-risk patients; however, additional CT scan and the risk of radiation have to be balanced against the cost and morbidity associated with retears that may require another surgical option rather than continuing the long-term rehabilitation, especially in younger patients.

## Abbreviations

AHI: Acromiohumeral interval
CT: Computed tomography
FE: Forward elevation
JOA: Japanese orthopaedic association
MRI: Magnetic resonance imaging
T-scale: Translation of the humeral head scale
UCLA: University of California–Los Angeles

## References

[1] Nam D, Maak TG, Raphael BS, Kepler CK, Cross MB, Warren RF (2012). Rotator cuff tear arthropathy: evaluation, diagnosis, and treatment: AAOS exhibit selection. J Bone Joint Surg Am.

[2] Gartsman GM (1997). Massive, irreparable tears of the rotator cuff. Results of operative debridement and subacromial decompression. J Bone Joint Surg Am.

[3] Duralde XA, Bair B (2005). Massive rotator cuff tears: the result of partial rotator cuff repair. J Shoulder Elb Surg.

[4] Hanusch BC, Goodchild L, Finn P, Rangan A (2009). Large and massive tears of the rotator cuff: functional outcome and integrity of the repair after a mini-open procedure. J Bone Joint Surg Br..

[5] Burkhart SS, Barth JR, Richards DP, Zlatkin MB, Larsen M (2007). Arthroscopic repair of massive rotator cuff tears with stage 3 and 4 fatty degeneration. Arthroscopy.

[6] Nada AN, Debnath UK, Robinson DA, Jordan C (2010). Treatment of massive rotator-cuff tears with a polyester ligament (Dacron) augmentation: clinical outcome. J Bone Joint Surg Br.

[7] Moursy M, Forstner R, Koller H, Resch H, Tauber M (2009). Latissimus dorsi tendon transfer for irreparable rotator cuff tears: a modified technique to improve tendon transfer integrity. J Bone Joint Surg Am.

[8] Glanzmann MC, Goldhahn J, Flury M, Schwyzer HK, Simmen BR (2010). Deltoid flap reconstruction for massive rotator cuff tears: mid- and long-term functional and structural results. J Shoulder Elb Surg.

[9] Miyoshi N, Suenaga N, Oizumi N, Taniguchi N, Ito H (2014). Rotator cuff reconstruction and humeral head replacement using smaller humeral prostheses in cuff tear arthropathy patients under 70 years of age. Open J Orthop.

[10] Goldberg SS, Bell JE, Kim HJ, Bak SF, Levine WN, Bigliani LU (2008). Hemiarthroplasty for the rotator cuff-deficient shoulder. J Bone Joint Surg Am.

[11] Boileau P, Gonzalez JF, Chuinard C, Bicknell R, Walch G (2009). Reverse total shoulder arthroplasty after failed rotator cuff surgery. J Shoulder Elb Surg.

[12] Galatz LM, Ball CM, Teefey SA, Middleton WD, Yamaguchi K (2004). The outcome and repair integrity of completely arthroscopically repaired large and massive rotator cuff tears. J Bone Joint Surg Am.

[13] Jost B, Pfirrmann CW, Gerber C, Switzerland Z (2000). Clinical outcome after structural failure of rotator cuff repairs. J Bone Joint Surg Am.

[14] Keener JD, Wei AS, Kim HM, Paxton ES, Teefey SA, Galatz LM (2010). Revision arthroscopic rotator cuff repair: repair integrity and clinical outcome. J Bone Joint Surg Am.

[15] Morisawa K, Yamashita K, Asami A, Nishikawa H, Watanabe H (1997). Spontaneous rupture of the deltoid muscle associated with massive tearing of the rotator cuff. J Shoulder Elb Surg.

[16] Galatz LM, Connor PM, Calfee RP, Hsu JC, Yamaguchi K (2003). Pectoralis major transfer for anterior-superior subluxation in massive rotator cuff insufficiency. J Shoulder Elb Surg.

[17] Ellman H, Hanker G, Bayer M (1986). Repair of the rotator cuff. End-result study of factors influencing reconstruction. J Bone Joint Surg Am.

[18] Hamada K, Fukuda H, Mikasa M, Kobayashi Y (1990). Roentgenographic findings in massive rotator cuff tears. A long-term observation. Clin Orthop Relat Res.

[19] Hamada K, Yamanaka K, Uchiyama Y, Mikasa T, Mikasa MA (2011). Radiographic classification of massive rotator cuff tear arthritis. Clin Orthop Relat Res.

[20] Sanchez-Sotelo J, Cofield RH, Rowland CM (2001). Shoulder hemiarthroplasty for glenohumeral arthritis associated with severe rotator cuff deficiency. J Bone Joint Surg Am.

[21] Taniguchi N, D’Lima DD, Suenaga N, Chosa EA. New scale measuring translation of the humeral head as a prognostic factor for the treatment of large and massive rotator cuff tears. J Shoulder Elb Surg. 2017; 10.1016/j.jse.2017.08.026 29056484

[22] Ellman H (1987). Arthroscopic subacromial decompression: analysis of one- to three-year results. Arthroscopy.

[23] DeOrio JK, Cofield RH (1984). Results of a second attempt at surgical repair of a failed initial rotator-cuff repair. J Bone Joint Surg Am.

[24] Smith CD, Alexander S, Hill AM, Huijsmans PE, Bull AM, Amis AA (2006). A biomechanical comparison of single and double-row fixation in arthroscopic rotator cuff repair. J Bone Joint Surg Am.

[25] Cho NS, Lee BG, Rhee YG (2011). Arthroscopic rotator cuff repair using a suture bridge technique: is the repair integrity actually maintained?. Am J Sports Med.

[26] Taniguchi N, Suenaga N, Oizumi N, Miyoshi N, Araki N, Chosa E (2014). Surface-holding repair: an original arthroscopic rotator cuff repair technique. J Shoulder Elb Surg.

[27] Yamaguchi H, Suenaga N, Oizumi N, Hosokawa Y, Kanaya F (2012). Will preoperative atrophy and fatty degeneration of the shoulder muscles improve after rotator cuff repair in patients with massive rotator cuff tears?. Adv Orthop.

[28] Yamaguchi H, Suenaga N, Oizumi N, Hosokawa Y, Kanaya F (2011). Open repair for massive rotator cuff tear with a modified transosseous-equivalent procedure: preliminary results at short-term follow-up. J Orthop Sci.

[29] Taniguchi N, Suenaga N, Oizumi N, Miyoshi N, Yamaguchi H, Inoue K (2015). Bone marrow stimulation at the footprint of arthroscopic surface-holding repair advances cuff repair integrity. J Shoulder Elb Surg.

[30] Sugaya H, Maeda K, Matsuki K, Moriishi J (2007). Repair integrity and functional outcome after arthroscopic double-row rotator cuff repair. A prospective outcome study. J Bone Joint Surg Am.

[31] Iannotti JP, McCarron J, Raymond CJ, Ricchetti ET, Abboud JA, Brems JJ (2010). Agreement study of radiographic classification of rotator cuff tear arthropathy. J Shoulder Elb Surg.

[32] Vastamaki M, Lohman M, Borgmastars N (2013). Rotator cuff integrity correlates with clinical and functional results at a minimum 16 years after open repair. Clin Orthop Relat Res.

[33] Bright AS, Torpey B, Magid D, Codd T, McFarland EG (1997). Reliability of radiographic evaluation for acromial morphology. Skelet Radiol.

[34] Hamid N, Omid R, Yamaguchi K, Steger-May K, Stobbs G, Keener JD (2012). Relationship of radiographic acromial characteristics and rotator cuff disease: a prospective investigation of clinical, radiographic, and sonographic findings. J Shoulder Elb Surg.

[35] Jacobson SR, Speer KP, Moor JT, Janda DH, Saddemi SR, MacDonald PB (1995). Reliability of radiographic assessment of acromial morphology. J Shoulder Elb Surg.

[36] Saupe N, Pfirrmann CW, Schmid MR, Jost B, Werner CM, Zanetti M (2006). Association between rotator cuff abnormalities and reduced acromiohumeral distance. AJR Am J Roentgenol.

[37] Werner CM, Conrad SJ, Meyer DC, Keller A, Hodler J, Gerber C (2008). Intermethod agreement and interobserver correlation of radiologic acromiohumeral distance measurements. J Shoulder Elb Surg.

[38] Keener JD, Galatz LM, Stobbs-Cucchi G, Patton R, Yamaguchi K (2014). Rehabilitation following arthroscopic rotator cuff repair: a prospective randomized trial of immobilization compared with early motion. J Bone Joint Surg Am.

